# Supplementary material for: Identification of Novel Mutations by Targeted NGS Panel in Patients with Hyperferritinemia
Source: Genes (Basel). 2021 Nov 9;12(11):1778. doi: 10.3390/genes12111778 (PMC8623017; doi:10.3390/genes12111778)
Supplement: Supplementary file 1 [file genes-12-01778-s001.zip › genes-1451461-supplementary.pdf]

Table S1. Genes and genomic regions containing variants of interest, included in the NGS panel.

GENES (SYMBOL)	REGION	ROLE IN IRON HOMEOSTASIS
Aconitase 1 (ACO1, IRP1)	Promoter, UTRs, EXON +/- 50 bp 5' e 3'	Iron sensing
Beta-2-microglobulin (B2M)	Promoter, UTRs, EXON +/- 50 bp 5' e 3'	HFE interaction
Bone morphogenetic protein 6 (BMP6)	Promoter, UTRs, EXON +/- 50 bp 5' e 3'	Hepcidin regulation
Congenital Dyserythropoietic Anemia, Type 1 (CDAN1)	Promoter, UTRs, EXON +/- 50 bp 5' e 3'	Erythopoiesis
Ceruloplasmin (CP)	Promoter, UTRs, EXON +/- 50 bp 5' e 3'	Ferroxidase
Cytocrome b reductase 1 (CYBRD1, DCYTB)	Promoter, UTRs, EXON +/- 50 bp 5' e 3'	Ferric reductase
Erytroferrone (FAM132B)	Promoter, UTRs, EXON +/- 50 bp 5' e 3'	Hepcidin regulation
Ferritin, heavy polypeptide 1 (FTH1)	Promoter, UTRs, EXON +/- 50 bp 5' e 3'	Iron storage
Ferritin, light polypeptide (FTL)	Promoter, UTRs, EXON +/- 50 bp 5' e 3'	Iron storage
Furin (FURIN)	Promoter, UTRs, EXON +/- 50 bp 5' e 3'	Hepcidin cleaving enzyme (activator)

Hepcidin (HAMP)	Promoter, UTRs, EXON +/- 50 bp 5' e 3'	Master regulator
Hephaestin (HEPH)	Promoter, UTRs, EXON +/- 50 bp 5' e 3'	Ferroxidase
HFE	Promoter, UTRs, EXON +/- 50 bp 5' e 3'	Hepcidin regulation – Iron sensing - Type 1 HH
Hemojuvelin (HFE2)	Promoter, UTRs, EXON +/- 50 bp 5' e 3'	Hepcidin regulation – Type 2 HH
Hypoxia inducible factor 1 alfa (HIF1A)	Promoter, UTRs, EXON +/- 50 bp 5' e 3'	Hypoxia sensing
Haptoglobin (HP)	Promoter, UTRs, EXON +/- 50 bp 5' e 3'	Hemoglobin transporter
Iron-responsive element binding protein 2 (IREB2)	Promoter, UTRs, EXON +/- 50 bp 5' e 3'	Iron sensing
Neogenin (NEO1)	Promoter, UTRs, EXON +/- 50 bp 5' e 3'	Hepcidin regulation
Scavenger receptor class A member 5 (SCARA5)	Promoter, UTRs, EXON +/- 50 bp 5' e 3'	Ferritin receptor?
Serpin family A member 1 (SERPINA1)	Promoter, UTRs, EXON +/- 50 bp 5' e 3'	TMPRSS6 inhibitor[35]
Solute carrier family 11 member 2 (SLC11A2 – DMT1)	Promoter, UTRs, EXON +/- 50 bp 5' e 3'	Iron import

Solute carrier family 40 member 1 (SLC40A1 - FPN1)	Promoter, UTRs, EXON +/- 50 bp 5' e 3'	Iron export/ Type 4 HH/Ferroportin Disease
Transferrin (TF)	Promoter, UTRs, EXON +/- 50 bp 5' e 3'	Iron transport
Transferrin receptor 2 (TFR2)	Promoter, UTRs, EXON +/- 50 bp 5' e 3'	Hepcidin regulation – Iron sensing - Type 3 HH
Transferrin receptor 1 (TFRC)	Promoter, UTRs, EXON +/- 50 bp 5' e 3'	Iron import
Transmembrane protease, serine 6 (TMPRSS6)	Promoter, UTRs, EXON +/- 50 bp 5' e 3'	Hepcidin regulation/ IRIDA
rs11558492 (GNPAT)	chr1:231407741-231408441	Putative HH Modifier[49]
rs236918 (PCSK7)	chr11:117,091,359-117,091,859	Putative HH Modifier[31]
rs651007 (ABO)	chr9:136,153,625-136,154,125	Putative HH Modifier[50]
rs6486121 (ARNTL)	chr11:13,355,520-13,356,020	Putative HH Modifier[50]

rs174577 (FADS2)	chr11:61,604,564-61,605,064	Putative HH Modifier[50]
rs4921915 (NAT2)	chr8:18,272,216-18,272,716	Putative HH Modifier[50]

UTRs: Untraslated regions; HH: Hereditary Hemochromatosis; IRIDA: Iron Refractory Iron Deficiency Anemia; GNPAT: glyceronephosphate O-acyltransferase; PCSK7: Proprotein convertase 7; ABO: Alpha 1-3-N-acetylgalactosaminyltransferase and alpha 1-3-galactosyltransferase; ARNTL: Aryl hydrocarbon receptor nuclear translocator like; FADS2: Fatty acid desaturase 2; NAT2: N-acetyltransferase 2.

Table S2. List of 51 exonic non-synonymous variants and their allelic frequencies in the whole group of 36 patients and in subgroups (TSAT <45% and >45%), compared to gnomAD.

Gene	Amino Acid Change	dbSNP	allelic frequency (all)	allelic frequency (TSAT<45%)	allelic frequency (TSAT≥45%)	allelic frequency (gnomAD)	p (all vs gnomAD)
ACO1	p.Val498Met	rs375879049	0.014	0.000	0.045	0.000	ns
ACVR1	p.Ala15Gly	rs13406336	0.028	0.020	0.045	0.009	ns
BMP2	p.Ser37Ala	rs2273073	0.056	0.080	0.000	0.000	<0.001
BMP6	p.Leu96Pro	rs200573175	0.028	0.040	0.000	0.006	0.01
BMPR2	p.Ser775Asn	rs2228545	0.069	0.060	0.091	0.036	ns
CDAN1	p.Gly657Ser	rs61747153	0.014	0.020	0.000	0.004	ns
CDAN1	p.Arg891Cys	rs8023524	0.111	0.120	0.091	0.186	ns
CDAN1	p.Thr996Ser	rs754811446	0.014	0.000	0.045	0.000	ns
CP	p.Pro477Leu	rs35331711	0.014	0.020	0.000	0.004	ns
CP	p.Thr551Ile	rs61733458	0.045	0.060	0.000	0.031	ns
CP	p.Pro876Ser	rs767188986	0.014	0.020	0.000	0.000	ns
ERFE	p.Arg72Cys	rs1395008564	0.014	0.020	0.000	0.000	ns
ERFE	p.Pro117Leu	rs749538301	0.014	0.020	0.000	0.000	ns
ERFE	p.Gly196Arg	rs1247409936	0.014	0.020	0.000	0.000	ns
ERFE	p.Ala260Ser	rs111241405	0.014	0.000	0.045	0.020	ns
ERFE	p.Gly268Arg	rs139530631	0.014	0.020	0.000	0.036	ns

ERFE	p.Val185Met	rs117194634	0.014	0.000	0.045	0.013	ns
FTH1	p.Lys54Arg	rs186448909	0.014	0.020	0.000	0.003	ns
HAMP	P.Arg59Pro	novel	0.014	0.000	0.045	//	//
HAMP	p.Lys83delinsLeuIleTyrSerCysCysProArg	rs1159254691	0.014	0.020	0.000	0.000	ns
HFE	p.His63Asp	rs1799945	0.222	0.220	0.227	0.144	0.003
HFE	p.Ser65Cys	rs1800730	0.028	0.020	0.045	0.015	ns
HFE	p.Cys282Tyr	rs1800562	0.014	0.000	0.045	0.058	ns
HFE	p.Val295Ala	rs143175220	0.014	0.000	0.045	0.000	ns
HJV	p.Cys89Arg	novel	0.014	0.000	0.045	//	//
HJV	delezione	novel	0.014	0.000	0.045	//	//
HIF1A	p.Ala475Ser	rs138451482	0.014	0.000	0.045	0.001	ns
HIF1A	p.Pro582Ser	rs11549465	0.097	0.080	0.136	0.105	ns
PCSK7	p.Thr182Met	rs150784623	0.014	0.000	0.045	0.000	ns
PDGFB	p.Thr202Met	rs114786489	0.014	0.020	0.000	0.006	ns
PDGFB	p.Gly53Arg	rs148252800	0.014	0.020	0.000	0.001	ns
SERPINA1	p.Asp280Val	rs120912714	0.014	0.020	0.000	0.001	ns
SERPINA1	p.Thr292Ile	rs745624643	0.014	0.000	0.045	0.000	ns
SERPINA1	p.Glu366Lys	rs28929474	0.014	0.000	0.045	0.000	ns
SLC40A1	c.-205A>C	novel	0.014	0.020	0.000	//	//
SLC40A1	p.Val160Ala	novel	0.014	0.020	0.000	//	//
SLC40A1	p.Arg179Thr	rs765023388	0.014	0.000	0.045	0.00001	<0.001

SLC40A1	p.Ala350Asp	novel	0.014	0.020	0.000	//	//
SLC40A1	p.Gly494Asp	novel	0.014	0.020	0.000	//	//
SLC40A1	p.Val531Ala	novel	0.014	0.020	0.000	//	//
SMAD4	p.Asn188Asp	novel	0.014	0.020	0.000	//	//
SMAD7	p.Gly137Ala	rs757109163	0.028	0.000	0.045	0.000	ns
SMAD7	p.Gly39Arg	rs144504026	0.028	0.040	0.000	0.011	ns
TF	p.Gly150Ser	rs1799899	0.083	0.080	0.091	0.060	ns
TF	p.Pro462Ser	rs1049296	0.083	0.100	0.045	0.160	ns
TF	p.Gly544Glu	rs120918677	0.014	0.020	0.000	0.000	ns
TFR2	p.Leu224Arg	novel	0.014	0.000	0.045	//	//
TFR2	p.Arg455Gln	rs41303501	0.014	0.000	0.045	0.00342	ns
TFR2	p.Asp514Metfs12	novel	0.014	0.000	0.045	//	//
TFR2	p.Tyr547Phe	rs573769443	0.014	0.000	0.045	0.00009	<0.001
TFRC	p.Thr658Ala	rs748490018	0.014	0.000	0.045	0.000	ns

Table S3. Distribution of the variants predicted as probably damaging by *in silico* tools in each patient. Light grey denotes heterozygote carriers and dark grey homozygote carriers.

		ACO1	BMP6	CDAN1	CP	ERFE	HAMP	HFE	HFE	HFE	HIF1A	PCSK7	SERPINA1	SMAD7	TF	TF
	βTHAL	V498M	L96P	R891C	T551I	G196R	K83delins	H63D	S65C	C282Y	P582S	T182M	E366K	G39R	P462S	G544E
**1**				light grey												
**2**								light grey							light grey	
**3**											light grey					
**4**																
**5**															light grey	
**6**				light grey												
**7**																
**8**				light grey				dark grey								
**9**								dark grey								
**10**				light grey	light grey											
**11**																
**12**			light grey													
**13**																
**14**																
**15**									light grey						light grey	
**16**											light grey					
**17**						light grey					light grey			light grey	light grey	
**18**					light grey											
**19**				light grey				light grey								
**20**																
**21**																
**22**			light grey		light grey									light grey		
**23**											light grey					
**24**				light grey				light grey								light grey
**25**								dark grey							light grey	
**26**																
**27**							light grey				light grey					
**28**																
**29**	dark grey			light grey				light grey							light grey	

**30**															
**31**															
**32**															
**33**															
**34**															
**35**															
**36**															
